# Comprehensive genomic profiling and therapeutic implications for Taiwanese patients with treatment‐naïve breast cancer

**DOI:** 10.1002/cam4.7384

**Published:** 2024-06-19

**Authors:** Shang‐Hung Chen, Ka‐Po Tse, Yen‐Jung Lu, Shu‐Jen Chen, Yu‐Feng Tian, Kien Thiam Tan, Chien‐Feng Li

**Affiliations:** ^1^ National Institute of Cancer Research, National Health Research Institutes Tainan Taiwan; ^2^ Department of Oncology National Cheng Kung University Hospital, College of Medicine, National Cheng Kung University Tainan Taiwan; ^3^ ACT Genomics, Co. Ltd. Taipei Taiwan; ^4^ Division of Colorectal Surgery, Department of Surgery Chi Mei Medical Center Tainan Taiwan; ^5^ Department of Health and Nutrition Chia‐Nan University of Pharmacy and Science Tainan Taiwan; ^6^ Anbogen Therapeutics, Inc. Taipei Taiwan; ^7^ Department of Medical Research Chi Mei Medical Center Tainan Taiwan; ^8^ Institute of Precision Medicine National Sun Yat‐Sen University Kaohsiung Taiwan; ^9^ Department of Clinical Pathology and Laboratory Medicine Chi Mei Medical Center Tainan Taiwan; ^10^ Trans‐omic Laboratory for Precision Medicine Chi Mei Medical Center Tainan Taiwan

**Keywords:** breast cancer, comprehensive genomic profiling, next‐generation sequencing, precision medicine

## Abstract

**Background:**

Breast cancer is a heterogeneous disease categorized based on molecular characteristics, including hormone receptor (HR) and human epidermal growth factor receptor 2 (HER2) expression levels. The emergence of profiling technology has revealed multiple driver genomic alterations within each breast cancer subtype, serving as biomarkers to predict treatment outcomes. This study aimed to explore the genomic landscape of breast cancer in the Taiwanese population through comprehensive genomic profiling (CGP) and identify diagnostic and predictive biomarkers.

**Methods:**

Targeted next‐generation sequencing‐based CGP was performed on 116 archived Taiwanese breast cancer specimens, assessing genomic alterations (GAs), including single nucleotide variants, copy number variants, fusion genes, tumor mutation burden (TMB), and microsatellite instability (MSI) status. Predictive variants for FDA‐approved therapies were evaluated within each subtype.

**Results:**

In the cohort, frequent mutations included *PIK3CA* (39.7%), *TP53* (36.2%), *KMT2C* (9.5%), *GATA3* (8.6%), and *SF3B1* (6.9%). All subtypes had low TMB, with no MSI‐H tumors. Among HR + HER2− patients, 42% (27/65) harbored activating *PIK3CA* mutations, implying potential sensitivity to PI3K inhibitors and resistance to endocrine therapies. HR + HER2− patients exhibited intrinsic hormonal resistance via *FGFR1* gene gain/amplification (15%), exclusive of PI3K/AKT pathway alterations. Aberrations in the PI3K/AKT/mTOR and FGFR pathways were implicated in chemoresistance, with a 52.9% involvement in triple‐negative breast cancer. In HER2+ tumors, 50% harbored GAs potentially conferring resistance to anti‐HER2 therapies, including *PIK3CA* mutations (32%), *MAP3K1* (2.9%), *NF1* (2.9%), and copy number gain/amplification of *FGFR1* (18%), *FGFR3* (2.9%), *EGFR* (2.9%), and *AKT2* (2.9%).

**Conclusion:**

This study presents CGP findings for treatment‐naïve Taiwanese breast cancer, emphasizing its value in routine breast cancer management, disease classification, and treatment selection.

## Graphical abstract

This study offered the comprehensive genomic profiling (CGP) of treatment‐naïve breast cancer in the Taiwanese population. It underscored the advantages of integrating CGP data into routine breast cancer management, providing valuable insights into disease classification and treatment selection.

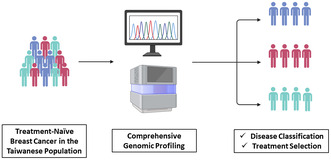

## 1 INTRODUCTION

Breast cancer stands as the most widespread malignant disease affecting women globally. In the year 2018, approximately 2 million new cases of breast cancer were diagnosed, resulting in 0.6 million fatalities worldwide.^1^ While the incidence of breast cancer is notably higher in Western nations, a striking surge in the occurrence rates among Asian populations, including China, Japan, South Korea, Singapore, and Taiwan, has been observed in recent decades.^2^, ^3^ Notably, Asian patients with breast cancer tend to be younger and face a less favorable prognosis compared with their Western counterparts.^2^, ^4^, ^5^ Until recently, precision medicine, which customizes treatment based on the specific molecular characteristics or genomic profiles of patients, has emerged as a promising strategy for cancer treatment. However, there is a scarcity of studies exploring the genomic background of breast tumors in the Asian population, leaving a significant gap in understanding the genomic and molecular attributes of Asian breast cancer.

Breast cancer is known as a heterogeneous disease with diverse biological behaviors and clinical treatment outcomes.^6^, ^7^ Several genomic‐based assays, including the Oncotype DX assay,^8^ MammaPrint,^9^ and PAM50,^10^ have been devised to predict responses to adjuvant therapy and the risk of distant recurrence in early‐stage hormone receptor (HR)‐positive breast cancer patients. Additionally, novel therapeutic agents targeting frequently mutated genes like *PIK3CA* or *BRCA1/2* have been developed and approved for treating patients with human epidermal growth factor 2 (HER2)‐negative breast cancer.^11^, ^12^ Next‐generation sequencing (NGS)‐based comprehensive genomic profiling (CGP) is a robust method for assessing various genomic aspects of tumor cells efficiently in terms of time and tissue usage.^13^, ^14^, ^15^ The highly sensitive and high‐throughput sequencing techniques can identify genomic alterations with as low as 5% mutant allele frequencies, enabling clinicians to categorize patients for different treatment strategies.^16^, ^17^, ^18^

This study was conducted to evaluate the clinical applicability of NGS‐based CGP in managing Taiwanese patients with breast cancer. We explored the genomic profiles and driver mutations within 116 treatment‐naive tumors from Chi‐Mei Medical Center (CMMC), assessing alterations in 440 cancer‐related genes. Our findings were then compared with a Western counterpart represented by the Memorial Sloan Kettering Cancer Center (MSKCC) cohort.^19^ Furthermore, we identified genomic alterations that could predict responses to various targeted therapies or were linked to prognosis. All of these outcomes underscore the advantages of integrating NGS into routine breast cancer management.

## 2 MATERIALS AND METHODS

### 2.1 Patients and tumor samples

We enrolled a total of 116 patients diagnosed with breast cancer (stage I–IV) according to the American Joint Committee on Cancer 7th edition in this study.^20^ All patients were treated in CMMC between February 2013 and October 2019, and tissues were collected during routine biopsy or resection before any systemic therapy. They were enrolled in the biobank after informed consent was obtained from all patients. The institutional review board approved collecting and using all specimens in this study of CMMC (IRB No. 10707004). De‐identified clinical information like TNM‐staging, histopathological results, follow‐up data were provided by the biobank.

### 2.2 Evaluation of HR and HER2 status

Cancer subgroups were classified according to the expression levels of the estrogen receptor (ER), progesterone receptor (PR), and HER2 proteins. The expression of these proteins was analyzed by immunohistochemical staining (IHC) on the formalin‐fixed paraffin‐embedded (FFPE) tissue section. Two independent pathologists evaluated the staining results. For ER and PR expression assessment, samples with >1% positively stained nuclei were defined as positive. HER2 expression scored 0, 1+, 2+, or 3+ based on the percentage and intensity of staining. HER2 score of 0 or 1+ was classified as negative, while 3+ was considered as positive. Samples with a score of 2+ were further examined by Ventana HER2 Dual in situ hybridization (ISH) assay (Roche Diagnostics), and those showing positive staining were classified as HER2‐positive.

### 2.3 Targeted sequencing and data analysis

Genomic DNA was extracted from FFPE tissue by using a QIAamp DNA FFPE tissue kit. Then DNA was quantified using the Quant‐iT dsDNA assay (Advanced Analytical Technologies, AATI) and quantitative real‐time PCR. A library was constructed using four pools of primer pairs targeting coding exons of 440 cancer‐related genes. Amplicons were ligated with barcoded adapters. The amplified library's quality and quantity were determined using the fragment analyzer (AATI) and Qubit (Invitrogen). Barcoded libraries were subsequently conjugated with sequencing beads by emulsion PCR and enriched by the Ion Chef System (Thermo Fisher Scientific) according to the Ion PI Hi‐Q Chef Kit protocol (Thermo Fisher Scientific) or Ion 540™ Kit Chef protocol (Thermo Fisher Scientific). Targeted sequencing was run on the Ion Proton or Ion S5 sequencer (Thermo Fisher Scientific). The mean depth is 500×, and uniformity at 100× was more than 75%.

Raw reads generated by the sequencer were mapped to the human reference genome (hg19) using the Ion Torrent Suite (version 5.10). Coverage depth was calculated using the Torrent Coverage Analysis plug‐in. Single nucleotide variants (SNVs) and small insertions and deletions (INDELs) were identified using the Torrent Variant Caller plug‐in (version 5.10). Variants were annotated by Variant Effect Predictor (version 100) using the databases from ClinVar, COSMIC v.86, and Genome aggregation database r2.0.2. Variants with coverage ≥25, allele frequency ≥5%, and actionable hotspot variants with allele frequency ≥2% were retained for the data analysis.

For copy number variants (CNVs) detection, amplicons with read counts in the lowest fifth percentile of all detectable amplicons and amplicons with a variation coefficient greater than 0.3 were removed. The remaining amplicons were normalized to correct the pool design bias. ONCOCNV (an established method for calculating copy number aberrations in amplicon sequencing data) was applied to normalize total amplicon number, amplicon GC content, amplicon length, and technology‐related biases, followed by segmenting the sample with a gene‐aware model.^21^ The method was also used to establish the baseline of copy number variations from samples in ACT Genomics in‐house database. All sample preparation, sequencing, and analytics were performed in a College of American Pathologists‐accredited laboratory.

### 2.4 Microsatellite analysis

Sequence diversity of more than 500 genomic loci were selected to identify the status the microsatellite instability of the given sample. To estimate the variation of sequence features of clinical samples, a set of normal samples were included to build the baseline features of selected loci. To minimize the bias derived from sequencing error, we eliminated the loci with high variability across repeat sequences. About 400 genomic loci across the sequencing regions are then used to estimate the microsatellite instability levels of clinical samples. Genomic patterns of the selected loci of pan‐cancer samples of microsatellite stable cohort and of microsatellite instability cohort were applied to train the in‐house microsatellite model by machine learning. The microsatellite state (either stable or instability) of clinical samples will be identified by the in‐house model with the genomic features of the selected loci.

### 2.5 Tumor mutation burden calculation

Tumor mutation burden (TMB) is calculated using the sequenced regions of ACTOnco® + to estimate the number of somatic non‐synonymous mutations per megabase of all protein‐coding genes (whole exome). Somatic mutations are identified by two approaches: database approach and patient‐derived approach. The database approach matches each variant to records in public databases in the following order (1) Genome Aggregate (gnomAD, https://gnomad.broadinstitute.org/), (2) the Cancer Genome Atlas (TCGA), and (3) Catalog of Somatic Mutations in Cancer (COSMIC). Variants matched to records in gnomAD were designated as germline mutations, while variants matched to records in TCGA and COSMIC were designated as somatic mutations. Subsequently, a patient‐derived approach derived from Sun et al. and Hossein et al. was applied to the remaining variants.^22^, ^23^ This approach considers variant frequency, copy number variation, zygosity, and NGS tumor purity to assign somatic or germline status to each variant. With the number of somatic variants used, TMB can be calculated. Driver mutations were filtered out to decrease potential bias in the calculation. A regression‐based model is applied to correct for synonymous mutations and panel size. The TMB analysis has been benchmarked using TCGA data by the TMB Harmonization Project of Friends of Cancer Research.^24^ TMB is reported as “Cannot Be Determined” if the tumor purity of the sample is <30%.

### 2.6 Fusion gene test

According to the manufacturer's instructions, extracted RNA was reverse transcribed using the SuperScript VILO cDNA Synthesis Kit (Invitrogen). Synthesized cDNA was subjected to library construction using the Ion AmpliSeq HD Library Kit (Life Technologies). The quality and quantity of the amplified library were determined using the fragment analyzer (AATI) and Qubit (Invitrogen). Sequencing was performed on the Ion 540TM Chip/Ion P1TM Chip and Ion GeneStudio™ S5 Prime System/Ion Proton™ System (Life Technologies). All assays were performed following ACT Genomics testing SOPs.

### 2.7 Determination of oncogenicity and actionability

Putative oncogenic mutations were defined using the OncoKB driver annotation algorithms in cBioportal (https://www.cbioportal.org/oncoprinter). Level of actionability was determined based on the ESMO Scale of clinical actionability of molecular targets (ESCAT) definition.^25^

### 2.8 Statistical analysis

Descriptive statistics were summarized as a median for age and frequency distribution for categorical variables. Mann–Whitney *U*‐test was used to compare the difference in age distribution between cohorts, while nominal variables were compared using Pearson's chi‐squared test or Fisher's exact test. Disease‐free survival (DFS) was defined as the time between diagnosis of primary tumor and occurrence of metastasis or local recurrence. Survival analyses were analyzed by Kaplan–Meier curves and log‐rank test. All statistical analyses were two‐sided and performed using SPSS software ver. 23 (IBM SPSS Statistics, RRID: SCR_019096) and GraphPad Prism 8 for Windows (GraphPad Prism, RRID: SCR_002798). A *p*‐value ≤0.05 was considered statistically significant.

## 3 RESULTS

### 3.1 Patient and clinicopathological characteristics

**FIGURE 1:**
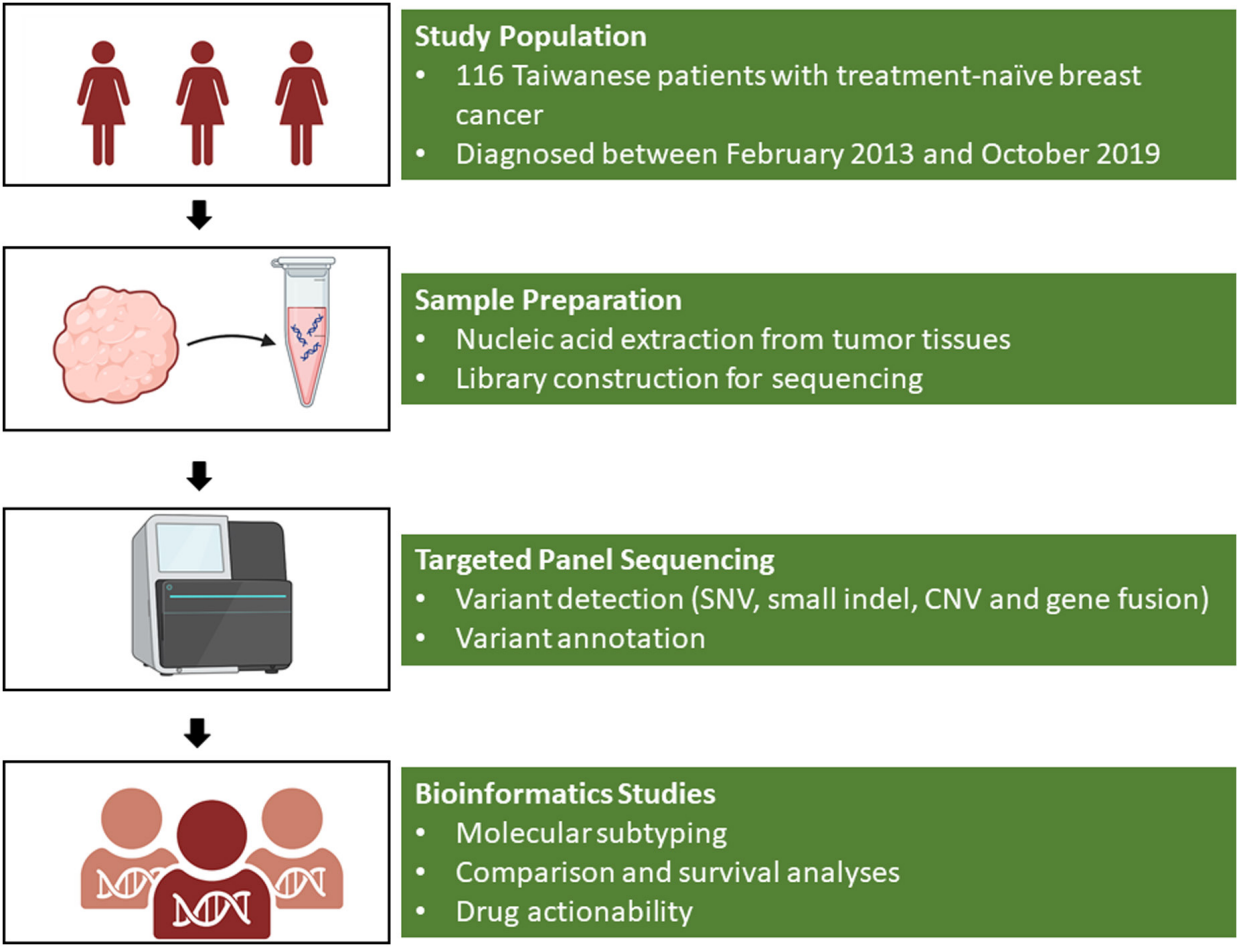
The study workflow investigating genomic profiling in Taiwanese patients with breast cancer.

The study workflow is presented in Figure 1. A total of 116 tumor tissues were collected from 116 breast cancer patients prior to any systemic treatment, and these tissues were subsequently analyzed. The characteristics of the patients can be found in Table 1. Out of the 116 cases, 82 (70.7%) were diagnosed with early‐stage disease (stage I and II), and the majority of them were diagnosed with invasive ductal carcinoma (IDC; *n* = 109, 94%). Based on the results of IHC staining, 80 out of the 116 (69%) were categorized as HR‐positive (HR+), with 77 (66.4%) and 72 (62%) being classified as ER‐positive or PR‐positive, respectively. Additionally, 34 (29.3%) were classified as HER2‐positive (HER2+). For the subsequent analyses, we further divided the patients into three subgroups based on the expression of HR and HER2: (1) HR + HER2− (*n* = 65, 56%), (2) HER2+ (*n* = 34, 12.9%), and (3) HR−HER2− or triple‐negative breast cancer (TNBC; *n* = 17, 14.7%).

**TABLE 1:** Demographics and clinicopathological characteristics of the CMMC cohort.

Characteristics	CMMC (*n* = 116)
	No. (%)
Age (median, range)	58 (25–89)
<50 years	35 (30.2)
≥50 years	81 (69.8)
Gender	
Female	116 (100)
Male	0 (0)
AJCC stage	
I	28 (24.1)
II	54 (46.6)
III	23 (19.8)
IV	7 (6)
Nil	4 (3.4)
Pathological type	
IDC	109 (94)
ILC	6 (5)
Others	1 (1)
ER status	
Positive	77 (66.4)
Negative	39 (33.6)
PR status	
Positive	72 (62)
Negative	44 (38)
HER2 status	
Positive	34 (29.3)
Negative	82 (70.7)
Ki67 index	
<20	79 (68.1)
≥20	37 (31.9)
Intrinsic subtypes	
HR + HER2−	65 (56)
HER2+	34 (29.3)
TNBC	17 (14.7)

Abbreviations: AJCC, The American Joint Committee on Cancer; ER, estrogen receptor; HER2, human epidermal growth factor receptor 2; HR, hormone receptor; IDC, invasive ductal carcinoma; ILC, invasive lobular carcinoma; LN, lymph node; PR, progesterone receptor; TNBC, triple‐negative breast cancer.

### 3.2 Genomic characteristics of Taiwanese breast cancers

**FIGURE 2:**
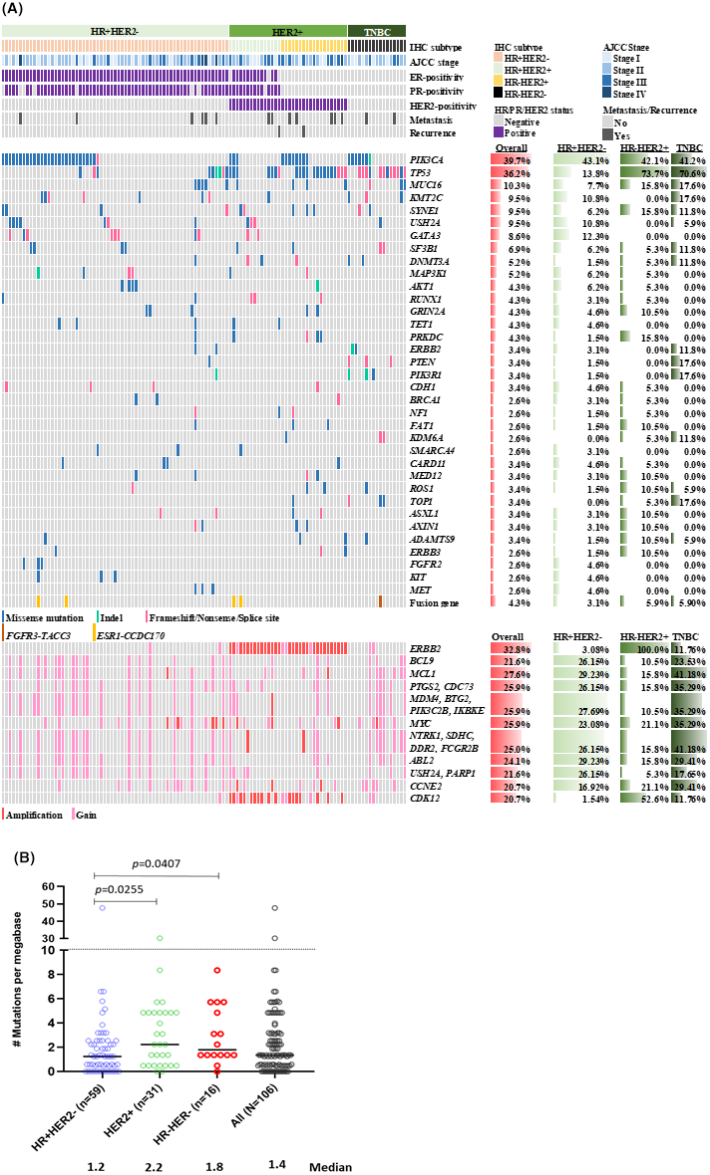
(A) Genomic landscape of the most frequently altered genes in Taiwanese breast cancer. Each column represents one sample and was sorted according to the IHC subtype, the stage of the disease. Different mutation types and copy number changes are labeled with colors in the central and lower panels. The names of the genes are marked on the left panel. The overall and within subgroups prevalence of mutations of each gene is shown on the right panel. AJCC, The American Joint Committee on Cancer; HER2, human epidermal growth factor receptor 2; HR, hormone receptor. (B) Comparison of TMB between different IHC subtypes in the CMMC cohort. TMB of 106 FFPE tumor tissues with adequate tumor cellularity are shown.

A total of 553 somatic mutations were identified in 116 tumor tissues, comprising 414 (74.9%) SNVs and 54 (9.8%) small Indels, as well as 1866 CNVs (Tables S1 and S2). The genomic landscape is depicted in Figure 2. In accordance with previous studies conducted in Western and Asian populations,^1^, ^2^, ^3^, ^4^, ^5^ the most frequently mutated driver genes in the entire cohort were *PIK3CA* (39.7%) and *TP53* (36.2%), followed by *KMT2C* (9.5%), *GATA3* (8.6%), *SF3B1* (6.9%), *DNMT3A* (5.2%), and *MAP3K1* (5.2%). Subgroup analysis revealed that the frequency of *PIK3CA* mutations remained consistent across the IHC subgroups. However, the prevalence of *TP53* mutations was lower in the HR + HER2− subgroup compared to the HER2+ and TNBC subgroups (13.8% vs. 61.8% and 70.6%, respectively). Notably, truncating mutations were the predominant *TP53* aberrations in the TNBC subgroup, accounting for 75% of cases (9 out of 12), while missense mutations in the DNA‐binding domain (DBD) were common alterations in other subtypes (Figure S1). Moreover, TNBC exhibited a higher mutation rate of *ERBB2* (11.8%), *PTEN* (17.6%), and *PIK3R1* (17.6%) in comparison to other subtypes. Conversely, *GATA3* mutations, primarily frameshift mutations on exon 5, were exclusively detectable in the non‐TNBC subgroups (HR + HER2− and HER2+).

In the CNA analysis, we observed *ERBB2* gene amplification or copy number gain in all tumors categorized as the HER2+ subgroup based on IHC results (Figure 2). Within the *ERBB2*‐amplified/gained tumors, concurrent amplification of *CDK12* (*n* = 22) was identified in 61.8% of cases. Additionally, our analysis of RNA‐derived fusion genes revealed the presence of four *ESR1‐CCDC170* fusions, two of which were detected in both HR + HER2− and HER2+ tumors. Furthermore, we found one *FGFR3‐TACC3 f*usion exclusively in the TNBC group (Figure 2).

TMB derived from panel sequencing is a widely recognized genomic marker used to predict the response to immune checkpoint inhibitor (ICI) treatments in solid tumors, including breast cancer.^26^, ^27^ The distribution of TMB, as depicted in Figure 2, revealed that all patient subsets in this cohort exhibited relatively lower TMB values (median = 1.4; ranging from 0 to 47.7 mutations per megabase [Mb]). Notably, TNBC and HER2+ tumors demonstrated significantly higher TMB levels compared with HR + HER2− tumors (1.8 and 2.2 vs. 1.2 mutations/Mb; *p* = 0.0407 and 0.0205, respectively). Despite the generally low mutational rate, two patients (1.7%) exhibited hyper‐mutation phenotypes, characterized by TMB values exceeding 10 mutations/Mb, with TMB counts of 47.7 and 30.2 mutations/Mb, respectively. Consistent with the TMB profile, all samples in our cohort exhibited microsatellite stable features, as confirmed by NGS data (Data not shown).

To investigate molecular factors contributing to inter‐ethnic differences in breast cancer incidence and aggressiveness, we compared the mutation frequencies of top‐ranked putative driver genes in the CMMC cohort with those in the publicly available MSKCC dataset. Overall, the prevalence of most putative driver mutations was similar across the two cohorts (Figure S2). However, mutations in *GATA3* (16% vs. 6%, *p* = 0.005), *MAP3K1* (12.5% vs. 2.6%, *p* = 0.0009), and *PTEN* (8.3% vs. 2.6%, *p* = 0.032) were significantly more prevalent in the MSKCC cohort, whereas the CMMC cohort exhibited a higher frequency of oncogenic mutations in *FGFR2* (2.6% vs. 0.2%, *p* = 0.013). These findings underscore the distinct genomic landscape of Taiwanese breast cancer.

### 3.3 Assessment of *ERBB2* and *PIK3CA* status

**FIGURE 3:**
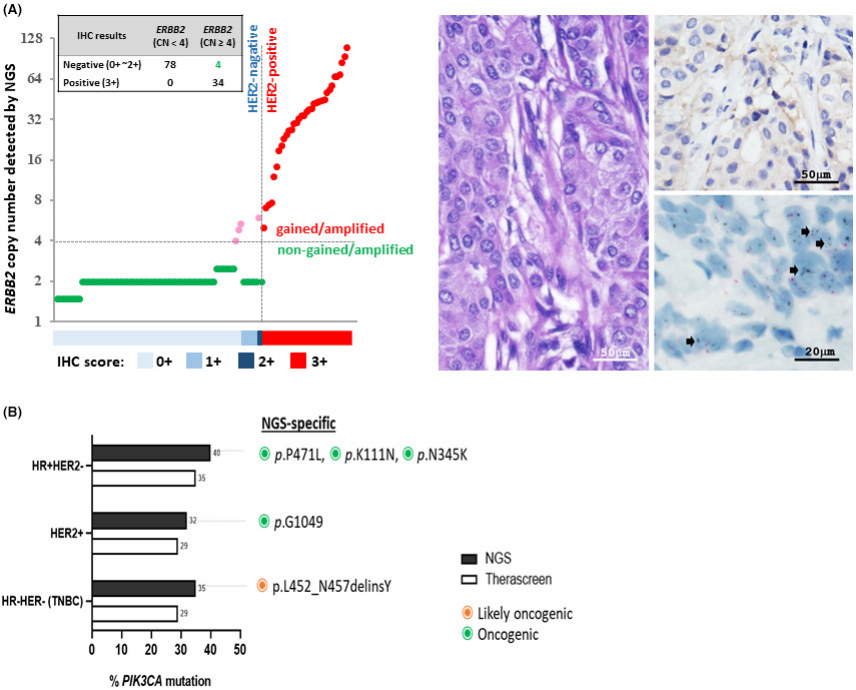
Concordance between NGS and other diagnostic methods. (A) The graph on the left shows the concordance between HER2 protein expression determined by IHC and *ERBB2* gene copy number estimated by NGS. HER2‐positive (IHC score ≥ 3+) and HER2‐negative cases (IHC score 0–2+) are indicated by red and green dots, respectively, while pink dots represent cases with discordant results in IHC staining and NGS that were further validated with Dual in Situ Hybridization (DISH) method. Representative images of one sample with inconsistent results (IHC score = 1+, CN estimated by NGS = 6) are shown on the right, with the *ERBB2* amplification status further assessed by DISH. The arrows indicate where the *ERBB2* cluster were detected. (B) Frequency and spectrum of *PIK3CA* activating mutations identified by NGS and FDA‐recognized companion diagnostic PCR Kit therascreen® in different IHC subgroups. Variants that were only detected by NGS platform were highlighted in green and orange.

HER2 protein overexpression or amplification of the *ERBB2* gene serves as a crucial predictive biomarker for identifying breast or gastric cancer patients who may benefit from HER2‐targeted therapies.^28^ In this study, our objective was to assess the concordance between *ERBB2* gene amplification and HER2 protein expression as measured by NGS and IHC, respectively. Among the 116 cases analyzed, 34 (29.3%) were categorized as HER2‐positive (IHC score = 3+). For NGS‐based targeted sequencing, we classified samples with an absolute *ERBB2* copy number of four or more as *ERBB2* copy number (CN) gained or amplified (when CN was greater than 8). According to this classification, 38 out of 116 cases (32.8%) were identified as *ERBB2* gene gained/amplified, with an overall concordance of 96.5% (112/116) with the IHC results (see Figure 3). Among the four cases with discordant results, three were scored as 0+ by the IHC test, and one was scored as 1+, while the NGS‐detected *ERBB2* copy numbers ranged from 4 to 6. To validate the *ERBB2* amplification status, we performed a ISH assay on these specimens, which detected *ERBB2* gene amplification in all four cases, thus confirming the NGS results.

Activating mutations in *PIK3CA* were the most prevalent oncogenic events observed in HR + HER2− breast cancer cases across various ethnic backgrounds.^29^ Alpelisib, an alpha‐selective phosphoinositide 3‐kinase (PI3K) inhibitor, gained approval for the treatment of HR + HER2− breast cancers with *PIK3CA* mutations, as identified through the US FDA‐approved therascreen® PIK3CA PCR companion diagnostic (CDx) test.^30^ A concordance analysis was conducted to compare the detection of PIK3CA positivity using two different technology platforms (correlation coefficient = 0.9; *p* < 0.001; Figure 3). Although the results demonstrated a high concordance rate, NGS identified more *PIK3CA* mutations (two in HR + HER2−, one in HER2+, and one in HR−HER2− tumors) compared with the therascreen® platform.

### 3.4 Strength of actionable genomic alterations

**FIGURE 4:**
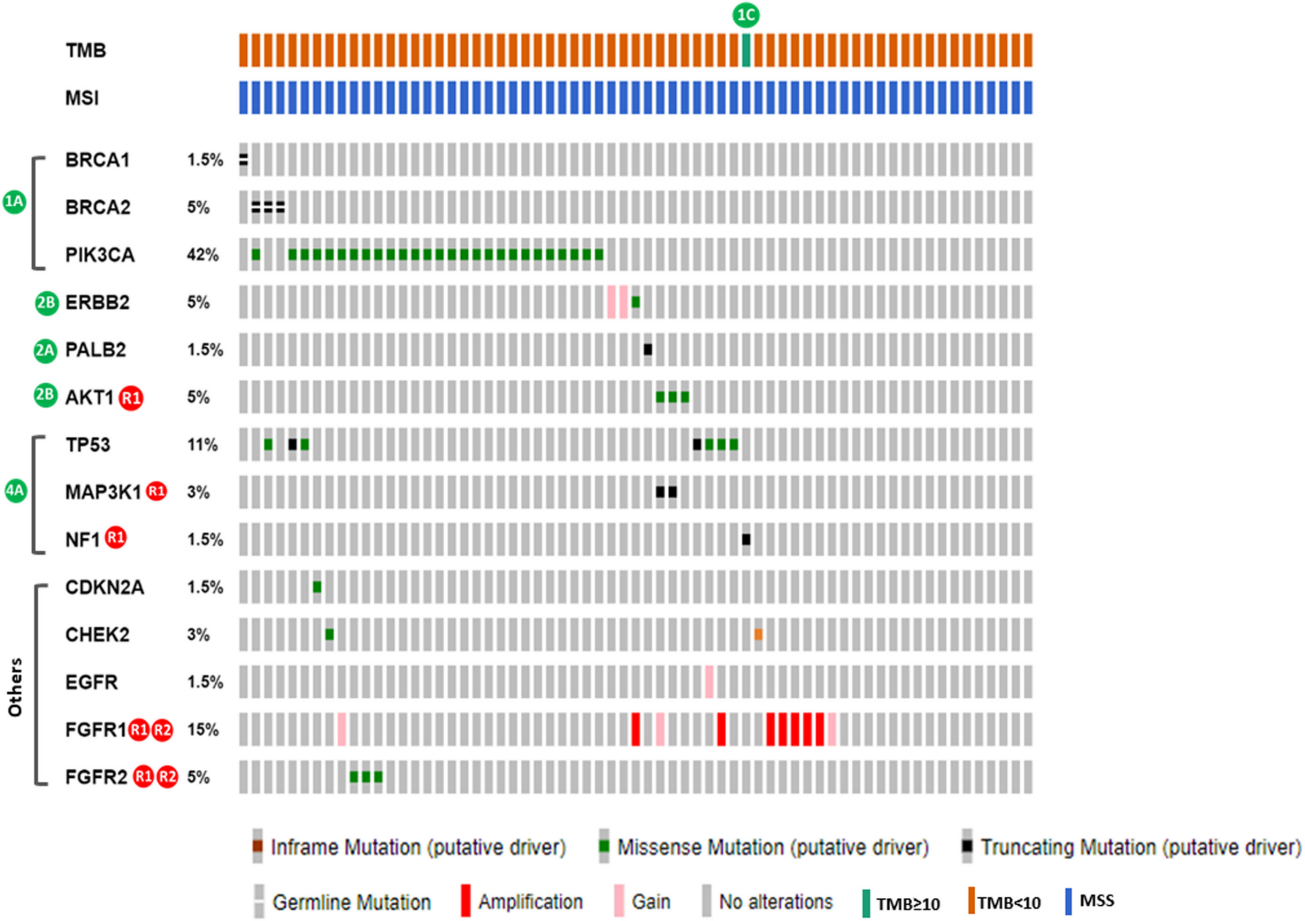
ESCAT‐annotated actionable mutations identified in HR + HER2− tumors.

To evaluate the efficacy of drugs tailored to oncogenic genomic alterations (GAs) specific to each breast cancer subtype, we gauged their potential actionability by assessing their strength through the ESCAT clinical evidence‐based criteria.^25^ Among 65 HR + HER2− patients, we identified 31 Tier 1A GAs, which encompassed 27 *PIK3CA* activating mutations, four *BRCA1/2* germline mutations, and one Tier 1C alteration with high TMB. These GAs predicted sensitivity to drugs targeting PI3K, PARP inhibitors, and ICIs, respectively (Figure 4; Table 2). Additionally, *PIK3CA* mutations not only suggested sensitivity to PI3K inhibition but were also closely linked to resistance against endocrine therapies, classifiable as ESCAT Tier R1 aberrations (Figure 4). In the HR + HER2− subgroup, another crucial intrinsic resistance mechanism was the gain/amplification of the *FGFR1* gene, which was implicated in resistance to hormonal (Tier R1) and PI3K (Tier R2) targeted treatments. Interestingly, two HR + HER2− cases with *ERBB2* copy number gain, possibly expressing HER2 protein, could potentially be categorized as Tier 1B alterations, making them responsive to HER2‐targeted therapies (Figure 4). Among HR + HER2− patients without Tier I GAs, five out of the remaining 32 harbored Tier 2A/B mutations in *ERBB2*, *PALB2*, or *AKT1* (Figure 4).

**TABLE 2:** Variant pathogenicity or deleteriousness and allelic status for (A) germline BRCA1/2, (B) germline and (C) somatic homologous recombination‐associated genes.

A				
Subtype	Gene	Amino acid change	Pathogenicity	Biallelic loss
HR + HER2−	*BRCA1*	p.Arg1443Ter	Pathogenic	Yes
HR + HER2−	*BRCA2*	p.Ser205PhefsTer9	Pathogenic	Yes
HR + HER2−	*BRCA2*	p.Leu1227GlnfsTer5	Pathogenic	No
HR + HER2−	*BRCA2*	p.Ser1722TyrfsTer4	Pathogenic	No
TNBC	*BRCA1*	c.5193 + 1G > T	Pathogenic	Yes
TNBC	*BRCA1*	p.Gln687Ter	Pathogenic	Yes
TNBC	*BRCA2*	p.Asp252ValfsTer24	Pathogenic	Yes

B				
Subtype	Gene	Amino acid change	Pathogenicity	Biallelic loss
HR + HER2−	*CHEK2*	c.847‐3_847‐2delTAinsGG	Pathogenic	Yes
HR + HER2−	*PALB2*	p.Lys149SerfsTer28	Pathogenic	CBD
HR + HER2−	*CHEK2*	p.His371Tyr	Pathogenic	No
HR + HER2−	*CHEK2*	p.Gly306Ala	Pathogenic	No
HER2+	*ATM*	p.Lys468GlufsTer18	Pathogenic	Yes
HER2+	*CHEK2*	p.His371Tyr	Pathogenic	Yes
HER2+	*CHEK2*	p.His371Tyr	Pathogenic	No
TNBC	*PTEN*	p.Leu57GlyfsTer5	Pathogenic	No
TNBC	*CHEK2*	p.His371Tyr	Pathogenic	No

C				
Subtype	Gene	Amino acid change	Deleteriousness	Biallelic loss
TNBC	*PTEN*	p.Leu320Ter	Deleterious	Yes
TNBC	*PTEN*	p.Tyr138PhefsTer41	Deleterious	No
TNBC	*PTEN*	p.Ala192HisfsTer7	Deleterious	No
TNBC	*PTEN*	p.Gln17Ter	Deleterious	No

Abbreviations: HER2, human epidermal growth factor receptor 2; HR, hormone receptor; TNBC, triple‐negative breast cancer.

CBD, cannot be determined.

**FIGURE 5:**
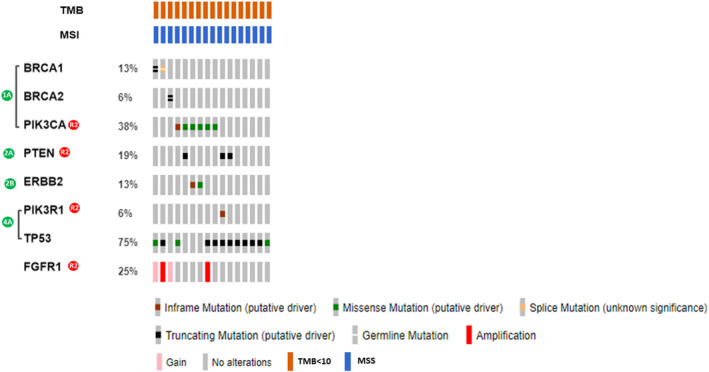
ESCAT‐annotated actionable mutations identified in TNBC.

**FIGURE 6:**
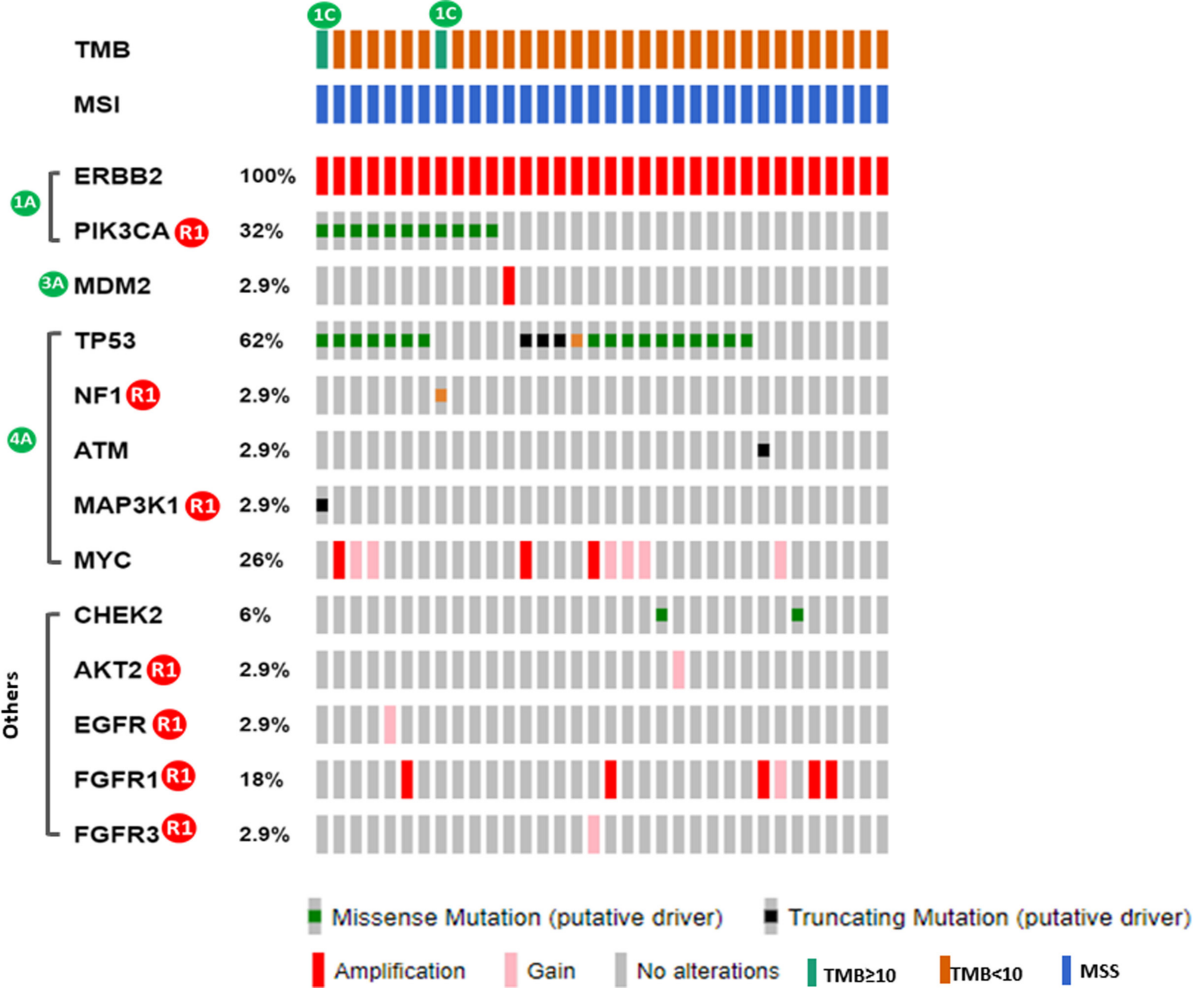
ESCAT‐annotated actionable mutations identified in HER2+ breast cancers.

In the TNBC subgroup, up to 19% of patients displayed Tier 1A *BRCA1/2* mutations, which was comparable to the HR + HER2− group (Figure 5; Table 2). Aberrant PI3K/AKT/mTOR and FGFR pathways were associated with chemoresistance and clinical outcomes in 52.9% and 25% of TNBC patients, respectively (Figure 5). In HER2+ breast cancers, approximately one‐third of patients had Tier R1 *PIK3CA* mutations, which were linked to resistance to anti‐HER2 therapy (Figure 6). Other GAs that could potentially confer resistance to anti‐HER2 therapies included mutations in *MAP3K1* (2.9%) and *NF1* (2.9%), as well as copy number gain/amplification of *FGFR1* (18%), *FGFR3* (2.9%), *EGFR* (2.9%), and *AKT2* (2.9%) genes (Figure 6). Across the entire cohort, we identified three Tier 2B *ERBB2* mutations, one (p.D769Y) in the HR + HER2− subgroup and two (p.L869R and p.Y772_A775dup) in the TNBC subgroup (see Figures 4 and 5; Figure S3).

Loss‐of‐function alterations or biallelic loss of homologous recombination repair (HRR) genes can result in homologous recombination deficiency (HRD), a surrogate biomarker that predicts sensitivity to PARP inhibitors.^31^ CN and zygosity data obtained from NGS can determine the presence of biallelic gene loss. In this study, 50% (2/4) of HR + HER2− and 100% (3/3) of TNBC patients with identified pathogenic germline *BRCA1/2* mutations exhibited biallelic loss (Table 2A). Furthermore, our analysis of HRR genes identified nine pathogenic germline mutations (including six *CHEK2*, one *PALB2*, one *ATM*, and one *PTEN*) and four deleterious somatic mutations (all four *PTEN*) within the entire cohort (Table 2B,C). All somatic HRR gene mutations were detected in TNBC subgroup. Among these assessable mutations, 37.5% (3/8) of the germline mutations and 25% (1/4) of the somatic mutations were identified as biallelic loss.

In addition to exploring genomic biomarkers predictive of drug response, we investigated intrinsic genomic alterations in early‐stage Taiwanese breast cancer. After excluding seven patients who were diagnosed with metastatic disease at presentation and one patient who died of a stroke shortly after diagnosis, 108 samples were included in the subsequent analyses. Eighteen of the 108 patients (16.7%) experienced relapse after initial treatment (53–71 months post‐surgery; Table S3). As illustrated in Figure S4, mutations in *TP53* and gains or amplifications in *RUNX1*, *UBR5*, *LYN*, and *CCNE1* were significantly associated with inferior DFS. These findings underscore the prognostic significance of these genomic alterations in Taiwanese breast cancer.

## 4 DISCUSSION

Accumulated research has provided genomic portraits of breast cancers from patients representing diverse ethnic backgrounds. However, the genomic data available from the Asian population with advanced breast cancer lesions primarily consists of events selected during tumor progression and the development of treatment resistance to neoadjuvant and adjuvant therapies. This study aimed to analyze the genomic profiles of 116 breast cancer specimens obtained from treatment‐naïve patients and assess the clinical relevance of NGS‐based CGP information. Our findings indicate that the most frequently mutated genes within our study cohort are in line with those reported in previous studies conducted in Western countries.^18^, ^19^ Prior studies have also indicated that the genomic landscape of breast cancer is largely similar between Asian and Western populations.^32^, ^33^, ^34^ The most notable differences between Asian and Western patients are observed in the HR + HER2− breast cancer subtype.^32^, ^33^ Alongside the findings of this study, such genomic comparisons could significantly enrich our comprehension of breast cancers in varied population groups.

NGS‐based CGP can help identify clinically significant druggable variants, offering insights into the prediction of susceptibility to currently approved targeted therapeutic approaches, such as *HER2* amplification and *PIK3CA* mutations. *ERBB2* gene amplification or protein overexpression serve as dependable biomarkers for guiding anti‐HER2 therapies in breast cancer. Clinical guidelines acknowledge several methods for evaluating HER2 status, such as IHC, fluorescence ISH, and bright field ISH.^35^ However, there can be discrepancies between IHC and ISH results, ranging from 1% to 12% in breast cancers.^35^, ^36^, ^37^ The high level of agreement found in our study suggests that HER2 status can also be accurately determined using NGS‐based CGP assays. This implies that NGS can offer supplementary genomic insights for molecular subtyping and treatment selection in breast cancer. Furthermore, our study's results demonstrate that 2.5% (3 out of 116) of Taiwanese patients with breast cancer had detectable targetable *ERBB2* activating mutations within the kinase domain. Identifying these actionable mutations, which can only be discerned through *ERBB2* gene sequencing, also underscores the significance of employing NGS‐based techniques in the care of patient with breast cancer.

Advancements in molecular diagnostic techniques have led to the exploration of biomarker‐driven therapeutic strategies for various cancer types, which have shown promising clinical benefits.^11^, ^12^ In the present study, we found that *PIK3CA* mutations were the most commonly observed alternations in Taiwanese patients with breast cancer, particularly in HR + HER2− breast tumors, accounting for 43.1% of cases. The results of SOLAR‐1 trial showed that breast cancer patients with hotspot mutations in the C2, helical, and kinase domains of *PI3K* (exons 7, 9, and 20) identified by the PCR‐based tool (therascreen PIK3CA RGQ PCR Kit), the US FDA‐approved CDx assay, exhibited superior clinical benefits from the combination therapy of alpelisib and fulvestrant.^11^ Prevalence of *PIK3CA* mutation identified by NGS in this cohort was consistent with those previously determined by other approaches.^38^, ^39^ However, NGS identified a substantial proportion of breast cancer patients carrying potentially oncogenic or oncogenic *PIK3CA* mutants that are not included in the therascreen® panel. This suggest that NGS can complement PCR‐based CDx tests, although the clinical relevance of some of these mutations remains to be investigated. Additionally, previous studies have reported the presence of double *PIK3CA* mutations in 12% to 15% of patients, which could potentially serve as biomarkers for high sensitivity to alpha‐specific PI3K inhibitors.^40^ In our present study, we identified three patients with HR + HER2− breast cancer harboring double oncogenic *PIK3CA* mutations using NGS‐based techniques and one patient using PCR‐based methods. As NGS‐based techniques can detect more cases of dual *PIK3CA* mutations, they may be a feasible diagnostic tool for identifying HR + HER2− breast cancer patients suitable for treatment with PI3K inhibitors.

PARP inhibitors, a type of emerging anticancer drug that targets poly (ADP‐ribose) polymerase, have received approval for various cancer types, including breast cancer.^41^, ^42^ Clinical trials have shown that PARP treatments, such as olaparib and talazoparib, can extend the survival of HER2‐negative breast cancer patients with germline *BRCA1/2* mutations.^12^, ^43^ Additionally, olaparib has gained approval from the US FDA for the adjuvant treatment of high‐risk, HER2‐negative breast cancer patients carrying germline *BRCA1/2* mutations who have undergone neoadjuvant or adjuvant chemotherapy.^44^ A pivotal trial involving 1836 patients with high‐risk, HER2‐negative early breast cancer and germline *BRCA1/2* variants demonstrated that those who received olaparib experienced an improvement in 3‐year disease‐free survival compared to the placebo group (87.5% vs. 80.4%; hazard ratio, 0.57; 99.5% confidence interval, 0.39 to 0.83; *p* < 0.001). Among various DNA repair mechanisms, the HRR system is crucial for precisely mending double‐strand breaks in DNA; the dysfunction of this repair system can have severe consequences for living cells.^41^, ^42^ PARP enzymes are responsible for repairing single‐strand breaks before DNA replication. Inhibiting them can lead to the formation of double‐strand breaks, which are lethal to cancer cells deficient in the HRR system. Mutations in HRR genes like *CHEK2*, *ATM*, *PALB2*, and *PTEN* can predict sensitivity to PARP inhibitors.^41^, ^42^ In the case of BRCAness tumors (found in breast, prostate, pancreatic, or ovarian cancer), most germline *BRCA1/2* mutants are biallelically inactivated, indicating their sensitivity to PARP inhibitors. However, somatic HRR gene deleterious mutations are often heterozygous, suggesting they may be bystander events and less likely to respond to PARP inhibitors. This complexity makes it challenging for clinicians to determine whether a tumor is primarily driven by HRR deficiency using clinical‐grade genomic assays. Nonetheless, PARP inhibitors may be considered for patients with HRR gene mutations who experience tumor relapse or distant metastases refractory to standard therapy.

In addition to identifying potential genomic biomarkers for tailoring targeted therapy, NGS‐based CGP can also detect genomic alterations associated with intrinsic resistance to endocrine and targeted therapies in breast cancer. *FGFRs* are frequently mutated in breast cancer, and their expression or mutation has been linked to disease progression and therapy resistance. For instance, aberrant FGFR signaling are not only associated with resistance to endocrine therapies but also resistance to CDK4/6 inhibitors.^45^ Our results demonstrated that *FGFR1/2/3* genomic alterations, primarily *FGFR1* amplification, were present in approximately 20% of HR + HER2− breast tumors, 23.8% of HER2+ breast tumors, and 24% of TNBC. Aberrant activation of the FGF/FGFR signaling cascade, resulting from gene amplification, activating mutations, and translocations of FGF or FGFR family members, was common in solid tumors, including breast cancer.^46^ In ER+ breast cancer, FGFR signaling aberrations have been reported to be involved in proliferation, metastasis, invasion, angiogenesis, and drug resistance.^47^ Although several FGFR‐targeting therapies, including erdafitinib, pemigatinib, and infigratinib, have gained approval from the US FDA for the treatment of FGFR‐mutated urothelial carcinoma and cholangiocarcinoma,^48^, ^49^, ^50^ their efficacy in treating breast cancer is still under investigation. A phase IIa study showed that patients with ER+ breast cancer who had progressed on aromatase inhibitors could benefit from the pan‐FGFR inhibitor AZD4547, regardless of their *FGFR1* amplification status or expression levels.^51^

Recently, ICIs have significantly transformed the treatment landscape for advanced‐stage malignancies characterized by high PD‐L1 expression, MSI‐high status, and high TMB.^26^, ^52^, ^53^, ^54^ TMB, which measures the number of somatic mutations in the tumor genome, is typically assessed through whole‐exome sequencing and is defined as the total number of mutations per coding area of the tumor genome.^26^ In contrast to other solid tumors, breast cancers usually exhibit a lower TMB; however, some retrospective studies have suggested that patients with high TMB breast cancer may experience enduring and complete responses to treatment regimens combining ICIs and chemotherapy.^55^, ^56^ Our study has revealed that NGS‐based targeted sequencing results can reliably infer the TMB and MSI status of breast cancer tissues. Within the HER2+ subgroup, two patients with exceptionally high TMB (47.6 and 30.2 mutations/Mb) tumors may be suitable candidates for ICI‐based treatment regimens.

Consistent with previous studies, our research identified *TP53* mutation as a prognostic indicator of reduced DFS. In most human cancers, two distinct types of *TP53* mutations are commonly observed. These mutations include those leading to the loss of function (LOFs), which hinder the anticancer activity, and those resulting in the gain of function (GOFs), which promote tumor growth.^57^ Furthermore, recent research has unveiled that truncating mutations of *TP53* can be categorized as separation‐of‐function mutations due to their distinct biochemical properties.^58^, ^59^ Functional studies have revealed that *TP53* truncating mutants located on exon‐6/exon‐7 can translocate to the mitochondria and enhance tumor characteristics by interacting with the mitochondria inner pore permeability regulator, Cyclophilin D (CypD). In the present study, our findings indicate that truncating variants are the predominant GAs of *TP53* in Taiwanese patients with TNBC.^60^ Given the limited treatment options for TNBC, further investigations into the effectiveness of CypD inhibitors, originally developed for other indications,^61^ in the treatment of TNBC are warranted.

The primary limitations of this cohort study are its retrospective nature and relatively small sample size. Additionally, the short follow‐up duration restricts the analysis of other prognostic factors associated with genetic information. Furthermore, we employed a commercially available cancer panel to investigate GAs, which means our examination was confined to the predefined genes within this specific cancer panel. Consequently, certain uncommon yet crucial genomic variations in breast cancer might remain undetected using targeted NGS techniques. Nonetheless, our findings do present potential actionable variants and therapeutic approaches for Taiwanese patients with breast cancer. Our study has indeed demonstrated the superior sensitivity of targeted panel NGS for detecting specific genomic variants, notably *HER2* amplifications and *PIK3CA* mutations. Additionally, the recent SAFIR02‐BREAST clinical trial has shown that CGP can improve outcomes for patients with metastatic breast cancer. In this prospective clinical trial, patients with ESCAT I/II genomic alterations experienced improved progression‐free survival when receiving genomics‐matched therapy.^62^ Moreover, the existing literatures support the cost‐effectiveness of genetics‐based approaches in reducing breast cancer risk.^63^, ^64^ With advancements in sequencing technologies leading to the identification of more drug targets and reduced sequencing costs, the cost‐effectiveness of CGP in clinical practice is expected to increase further. Consequently, the importance of further research to enhance the practical application of NGS‐CGP in the clinical setting is clear, highlighting the need for clinical trials that compare outcomes between CGP analyses performed on tumor tissue and circulating tumor DNA. The use of NGS for liquid biopsies could revolutionize the timely assessment of tumor evolution and the tailoring of treatment plans. Additionally, NGS technology is poised to revolutionize key fields such as single‐cell genomics, long‐read sequencing, epigenomics, and the integration of various omics technologies, paving the way for more comprehensive and individualized treatment approaches. The advent of real‐time sequencing and point‐of‐care testing promises to drastically change how quickly and effectively breast cancer is diagnosed and monitored. Moreover, advancements in bioinformatics and data analysis are crucial for interpreting the vast amounts of data generated by NGS. The integration of artificial intelligence is expected to greatly enhance the efficiency and accuracy of bioinformatic analyses, especially as the volume of data from multi‐omics research expands.

In summary, the use of CGP with NGS‐based targeted panels proves to be a viable and cost‐effective method for identifying clinically relevant genomic variants in breast cancer. This targeted NGS approach allows for simultaneous genomic profiling to inform treatment decisions in routine clinical practice and identify potential beneficial targeted therapies. Specifically, in the diagnosis of early‐stage HER2‐positive breast cancer, NGS techniques can not only validate IHC results but also assist in identifying patients with *ERBB2* activating mutations. Furthermore, NGS‐based CGP can aid in the early detection of *BRCA*‐mutated breast cancers, potentially opening the door to adjuvant treatment options with PARP inhibitors if germline variants are diagnosed. It's worth noting that significant genomic variants such as *PI3KCA*, *FGFR*, and TMB can also provide therapeutic insights. As a result, modern anticancer therapy demands multiple genomic tests, and our results underscore the clinical value of NGS‐based CGP for patients with breast cancer.

## AUTHOR CONTRIBUTIONS

**Shang‐Hung Chen:** Conceptualization (equal); validation (equal); writing – review and editing (equal). **Ka‐Po Tse:** Data curation (equal); formal analysis (equal); visualization (equal); writing – original draft (equal); writing – review and editing (equal). **Yen‐Jung Lu:** Investigation (equal). **Shu‐Jen Chen:** Conceptualization (equal); funding acquisition (equal); resources (equal). **Yu‐Feng Tian:** Investigation (equal). **Kien Thiam Tan:** Conceptualization (equal); data curation (equal); methodology (equal); project administration (equal); supervision (equal); writing – review and editing (equal). **Chien‐Feng Li:** Conceptualization (equal); methodology (equal); resources (equal); supervision (equal).

## FUNDING INFORMATION

This research was funded by the Health and Welfare Surcharge of Tobacco Products, the Health Promotion Administration, Ministry of Health and Welfare, Taiwan, grant number MOHW110‐TDU‐B‐212‐144,014.

## CONFLICT OF INTEREST STATEMENT

Ka‐Po Tse, Yen‐Jung Lu, Shu‐Jen Chen, and Kien Thiam Tan are employed by ACT Genomics Co., Ltd. Tumor NGS analysis for the study was supported by ACT Genomics Co., Ltd. Other authors have no conflict of interest with the content of the manuscript.

## ETHICS STATEMENT

The Ethics Committee and Institutional Review Board of Chi Mei Medical Center approved the study on July 20, 2018 (10707004) by using disconnected tumor samples/clinicopathologic information from the biobank following the ethical guidelines of the Helsinki Declaration and the regulations of our government.

## Supporting information


Figure S1:

Figure S2:

Figure S3:

Figure S4:

Table S1:

Table S2:

Table S3:

Data S1.

## Data Availability

Data is contained within the article or Supplementary Material.
