# Supplementary figures and images for: Comprehensive genomic profiling and therapeutic implications for Taiwanese patients with treatment‐naïve breast cancer
Source: Cancer Med. 2024 Jun 19;13(12):e7384. doi: 10.1002/cam4.7384 (PMC11187859; doi:10.1002/cam4.7384)

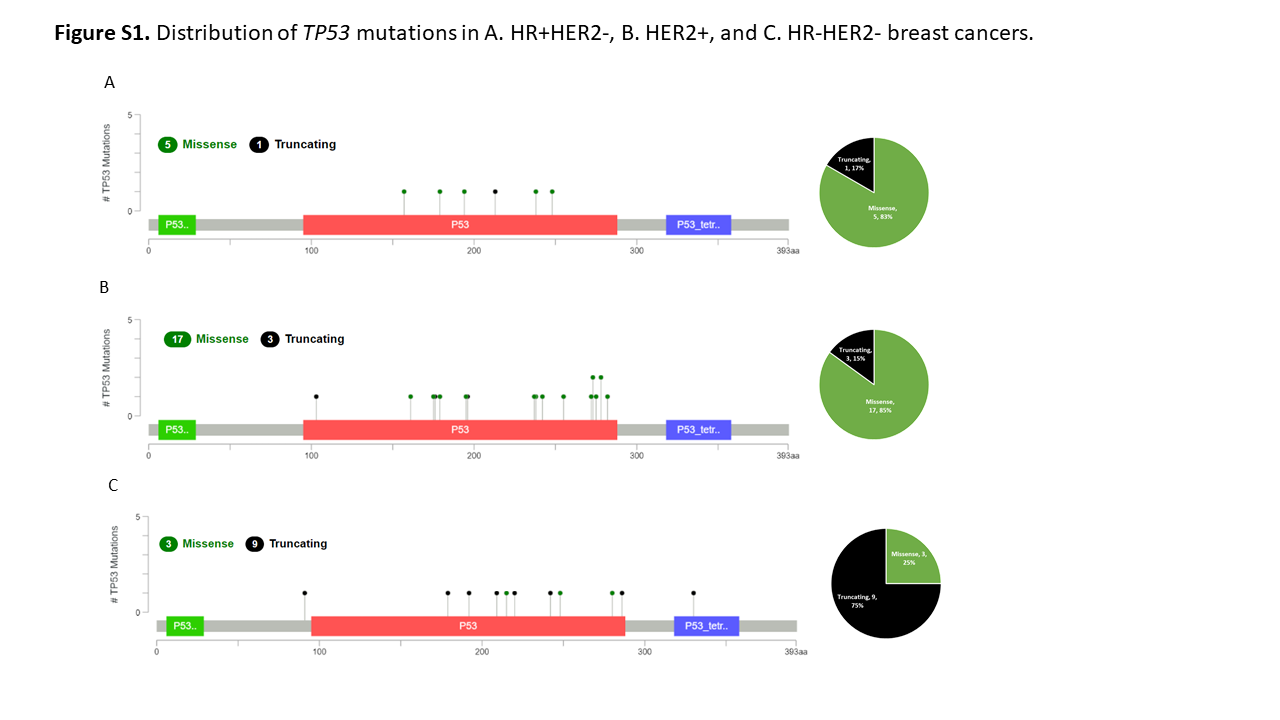

Supplement: Supplementary file 1 — Figure S1: [file CAM4-13-e7384-s006.tif]

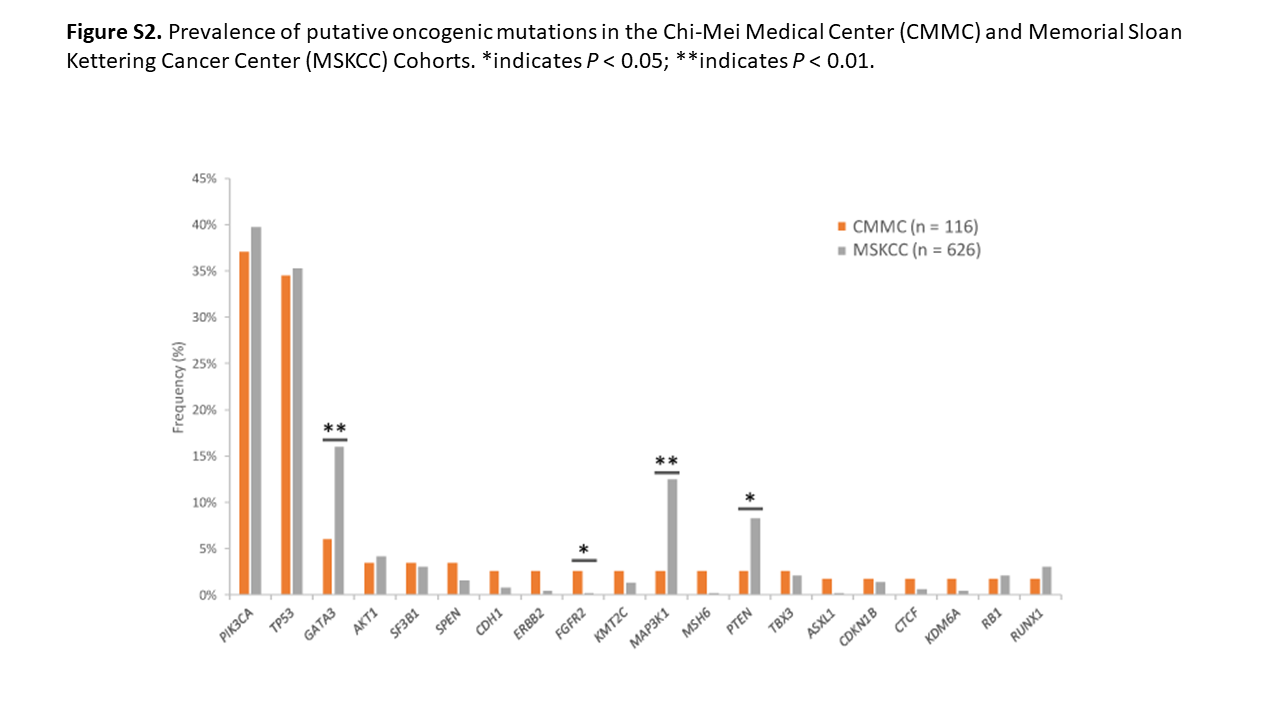

Supplement: Supplementary file 2 — Figure S2: [file CAM4-13-e7384-s005.tif]

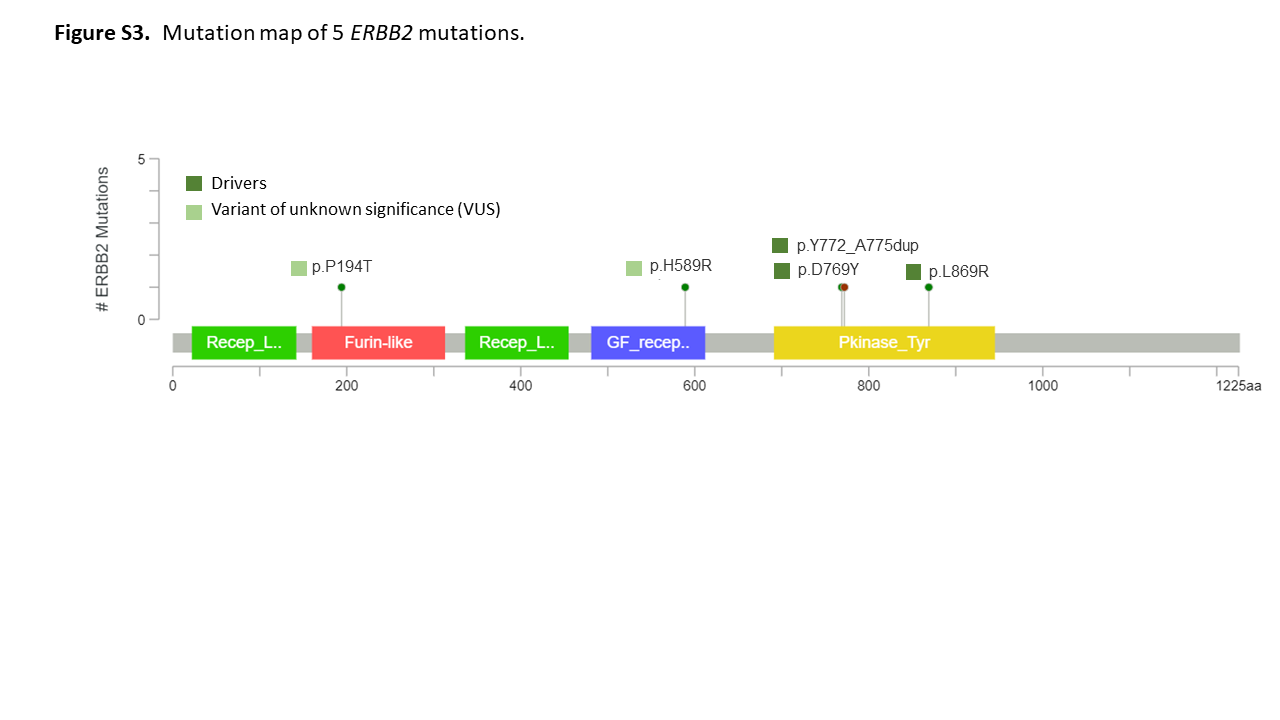

Supplement: Supplementary file 3 — Figure S3: [file CAM4-13-e7384-s001.tif]

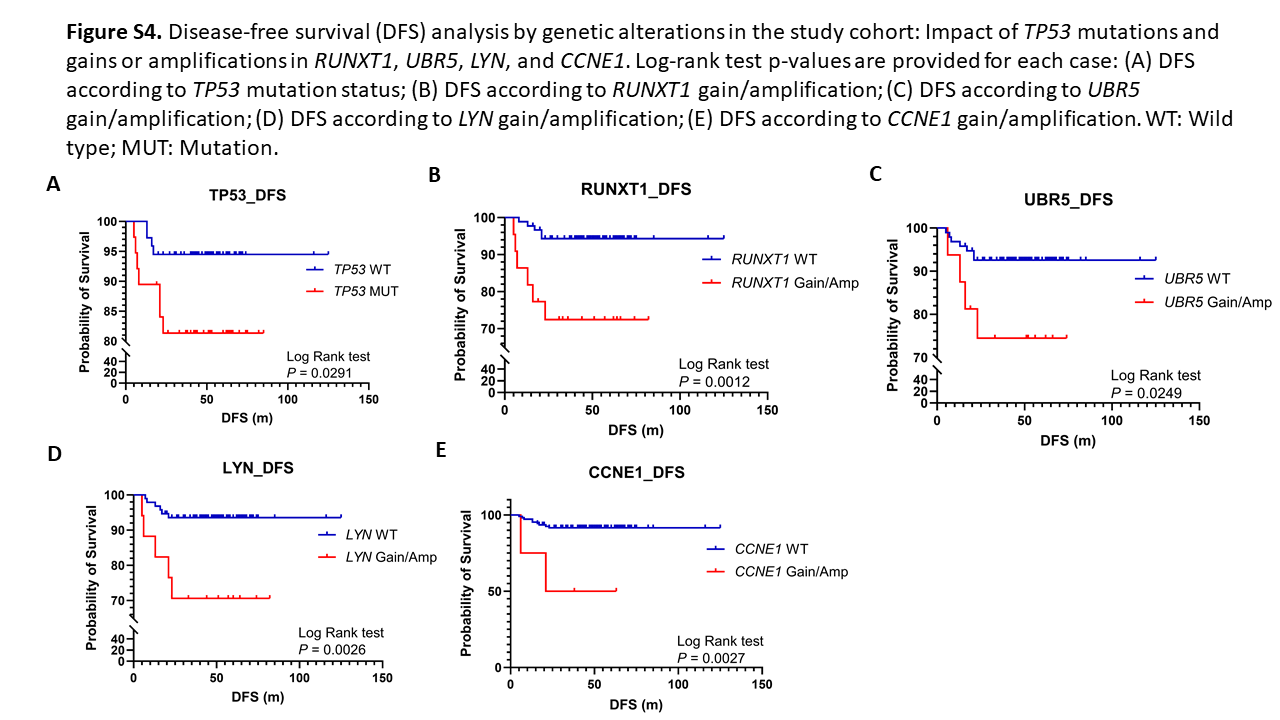

Supplement: Supplementary file 4 — Figure S4: [file CAM4-13-e7384-s004.tif]
