# Supplementary material for: Comprehensive genomic profiling and therapeutic implications for Taiwanese patients with treatment‐naïve breast cancer
Source: Cancer Med. 2024 Jun 19;13(12):e7384. doi: 10.1002/cam4.7384 (PMC11187859; doi:10.1002/cam4.7384)
Supplement: Supplementary file 8 — Data S1. [file CAM4-13-e7384-s008.docx]

Tables S1: SNVs of the CMMC breast cancer cohort, CNVs of the CMMC breast cancer cohort, and treatment outcomes of the CMMC breast cancer cohort.

Tables S2: SNVs of the CMMC breast cancer cohort, CNVs of the CMMC breast cancer cohort, and treatment outcomes of the CMMC breast cancer cohort.

Tables S3: SNVs of the CMMC breast cancer cohort, CNVs of the CMMC breast cancer cohort, and treatment outcomes of the CMMC breast cancer cohort.
